# Body image perception among Medical Students in Tunisia

**DOI:** 10.1192/j.eurpsy.2026.11542

**Published:** 2026-07-31

**Authors:** R. Ghammam, I. Houas, Y. Mkab, R. Hammami, Y. Jmal, I. Hamzaoui, S. Ben Fredj, I. Thlithi, F. Dhaghsni, M. Oueslati, H. ElMoucharref, C. Saliba, R. Attia, A. Chouchene, A. Skhiri, S. BenFredj

**Affiliations:** 1 Université de Sousse, Faculté de Médecine de Sousse; 2 Hôpital Universitaire Farhat Hached, Service de Médecine Préventive et Communautaire, LR19SP03; 3 Université de Sousse, Faculté de Médecine de Sousse, Développement Profesionnel 3; 4 Hôpital Universitaire Farhat Hached, Service de Médecine de travail, LR19SP03, Sousse, Tunisia

## Abstract

**Introduction:**

Body image is a fundamental aspect of identity and psychological well-being, shaped by social interactions, personal experiences, and environmental pressures. In today’s media-saturated world, unattainable sociocultural standards often, fuel body dissatisfaction. This dissatisfaction is a well-established risk factor for developing many mental health problems, a growing concern in global public health. Medical students face unique academic and professional stressors that may put them at a higher risk for these issues.

**Objectives:**

This study aims to explore the perceived body image among medical students in Tunisia.

**Methods:**

We carried out a cross-sectional study from 2024 to 2025 at the Faculty of Medicine of Sousse. We gathered data from a self-administered, anonymous online questionnaire, which was shared on official academic Facebook groups for each student year level. Body image concern was the primary outcome, measured with the Body Shape Questionnaire (BSQ). This standardized scale yields a score from 16 to 96, where higher scores indicate greater body image dissatisfaction.

**Results:**

A total of 243 students responded to the questionnaire. The majority of participants were female (69.5%). Among participants 62.96% lived away from their families, while 86.8% reported having a medium socio-economic status.

A marked concern about body image was observed in 12.8% of students and a moderate concern among 13.2% and a mil concern among 16.5% of students. Among students with moderate or marked Body shape concern (G1) 66.7% were female compared to 70.6 among the group with no or mild body shape concern (G2) with no significant difference (p=0.564). Among the first group 15.9% had a high socioeconomic level compared to 10.6% among the second group (p=0.263). Chronic Illness were reported by 17.5% of student with moderate or marked Body shape concern and 9.4% among students with no or mild body shape concern (p=0.086). No significant association between weight excess and body image concern among our population. However 84.1% of participant with moderate or marked Body shape concern had possible eating disorders compared to 41.1% among the second group with a significant association (p<0.010).

**Conclusions:**

Our findings indicate a significant prevalence of body image concern among medical students, affecting over 40% of the surveyed population. While gender, socioeconomic status, and chronic illness did not show a statistically significant association, the strong link between body image concern and a higher risk of eating disorders is a critical finding. These results highlight the need for mental health awareness campaigns and specific interventions tailored to address body image and eating disorder risks within student population.

**Disclosure of Interest:**

None Declared

